# The sponge genus *Ephydatia* from the high-latitude middle Eocene: environmental and evolutionary significance

**DOI:** 10.1007/s12542-016-0328-2

**Published:** 2016-09-28

**Authors:** Andrzej Pisera, Renata Manconi, Peter A. Siver, Alexander P. Wolfe

**Affiliations:** 1 Institute of Paleobiology, Polish Academy of Sciences, ul. Twarda 51/55, 00-818 Warsaw, Poland; 2 Dipartimento di Scienze della Natura e del Territorio (DIPNET), Università di Sassari, 07100 Sassari, Italy; 3 Botany Department, Connecticut College, New London, CT 06320 USA; 4 Department of Biological Sciences, University of Alberta, Edmonton, AB T6G 2E9 Canada

**Keywords:** Porifera, Freshwater sponges, Eocene, Canada, Climate, Morphological stasis, Porifera, Süßwasserschwämme, Eozän, Kanada, Klima, Morphologische Stasis

## Abstract

The freshwater sponge species *Ephydatia* cf. *facunda* Weltner, 1895 (Spongillida, Spongillidae) is reported for the first time as a fossil from middle Eocene lake sediments of the Giraffe kimberlite maar in northern Canada. The sponge is represented by birotule gemmuloscleres as well as oxea megascleres. Today, *E. facunda* inhabits warm-water bodies, so its presence in the Giraffe locality provides evidence of a warm climate at high latitudes during the middle Eocene. The morphological similarity of the birotules to modern conspecific forms suggests protracted morphological stasis, comparable to that reported for other siliceous microfossils from the same locality.

## Kurzfassung

Die rezente Süßwasserschwamm-Art *Ephydatia* cf. *facunda* Weltner, 1895 (Spongillida, Spongillidae) wird erstmals als Fossil aus den mittel-eozänen Seeablagerungen des Giraffe Kimberlit-Maars im nördlichen Kanada nachgewiesen. Die Schwammspezies ist sowohl durch birotule Gemmoskleren als auch durch Megaskleren (Oxen) belegt. Heute besiedelt *E. facunda* warm-temperierte Wasserkörper, somit spricht ihr Vorkommen für ein warmes Klima in hohen Breiten während des Mittel-Eozäns. Die morphologische Ähnlichkeit der Birotulen in Bezug auf moderne konspezifische Formen legt eine protrahierte morphologische Stasis nahe, vergleichbar mit derjenigen anderer kieseliger Mikrofossilien aus derselben Fundstelle.

## Introduction

Freshwater sponges (Porifera, Spongillida) are common in many modern continental waters, but their fossil record is somewhat sparse. Although marine sponges have existed since the Proterozoic (Pisera 2006; Van Soest et al. 2012), the earliest freshwater sponges do not appear in the fossil record until the Permo-Carboniferous of Europe (Schindler et al. 2008). Mesozoic occurrences of Spongillida are known from the USA during the Late Jurassic, and from England and Patagonia during the Lower Cretaceous (Pisera and Saez 2003). The oldest preserved gemmules (resting bodies of sponges) were found in the latter region (Chubut Valley) (Ott and Volkheimer 1972; Volkmer-Ribeiro and Reitner 1991). More common freshwater sponges have been discovered in Paleogene and Neogene deposits, with records originating from Germany, Siberia, Japan, Chile, and South Africa (see Pisera and Saez 2003). Recently, middle Eocene (~40 Ma) lake sediments within a kimberlite diatreme in northern Canada, referred to as the Giraffe locality, have yielded a rich assemblage of siliceous microfossils, including diatoms (Bacillariophyceae), chrysophytes (Chrysophyceae and Synurophyceae), euglyphids (Euglyphidae, Rhizaria), and spongillids (Pisera 2010; Siver et al. 2010; Pisera et al. 2013, 2014). Many of these forms have pronounced affinities with modern taxa (Siver and Wolfe 2005, 2009). The sponge microfossil record from the Giraffe locality comprises a wide array of loose spicules, including numerous megascleres, gemmuloscleres (spicules forming an armor for the resting bodies, and including both birotules and non-birotules), and microscleres. A new species, *Potamophloios canadensis*, belonging to the warm-water freshwater sponge family Potamolepiidae, was described by Pisera et al. (2013, 2014) from these sediments. In this paper, we report additional spicules from the Giraffe locality that belong unambiguously to the genus *Ephydatia* Lamouroux, 1816 (family Spongillidae).

## Geological setting

**Fig. 1:**
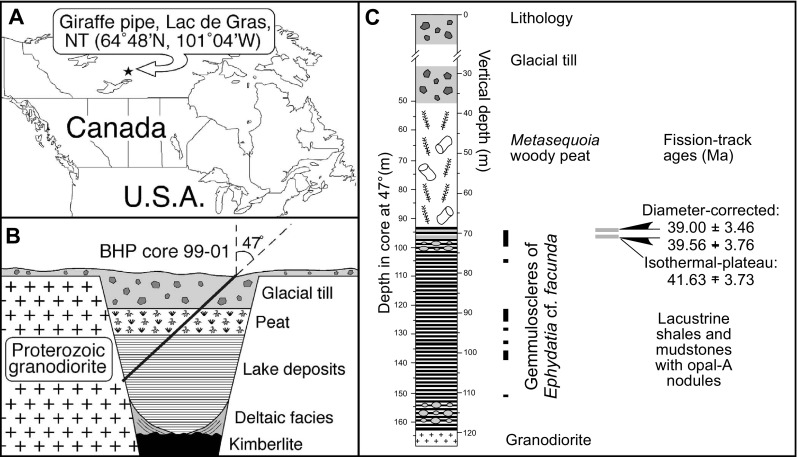
Location map and stratigraphy of the Giraffe fossil locality (northern Canada). **a** Location; **b** schematic stratigraphy; **c** lithostratigraphy and *Ephydatia* cf. *facunda* spicule occurrences (black vertical bars)

The Giraffe fossil locality (Wolfe et al. 2006; Siver and Wolfe 2009; Doria et al. 2011) is a kimberlite diatreme that was intruded into the Slave Craton of the Canadian Shield 47.8 ± 1.4 Ma ago and infilled with an organic sediment sequence that accumulated post-eruptively (Fig. 1). Over 60 m of lacustrine sediments (laminated shales and mudstones) comprise the lower maar facies, which is succeeded by ~40 m of terrestrial sediments that include significant amounts of *Metasequoia* foliage and wood. The transition between lacustrine and terrestrial sedimentation occurred ~38 Ma ago, based on glass fission-track ages (Doria et al. 2011), implying that the entire lake sequence is middle Eocene (Lutetian Stage) in age. The thermal and tectonic stability of the locality post kimberlite emplacement has resulted in excellent preservation of both siliceous and non-siliceous fossils (Wolfe et al. 2006; Doria et al. 2011).

## Materials and methods

The investigated material originates from a drill-core obtained in 1999 by BHP Billiton Diamonds Inc. during diamond exploration. The present study is based on isolated spicules obtained from core sediment samples (~200 mg) following treatment with 30 % H_2_O_2_, repeated washing with deionized water, and final suspension in reagent-grade propanol (isopropyl alcohol). The resulting suspensions were dried onto coverslips, attached with carbon glue to SEM stubs, sputter-coated with platinum, and examined with a Philips XL20 field-emission SEM (Institute of Paleobiology, Warsaw) at magnifications ranging from 100× to 5000×, using a voltage of 25 kV. Among the examined samples, those from the interval 97.39 m (71.23 m vertical equivalent depth) to 153.30 m (112.12 m vertical depth) in the core revealed the richest sponge assemblages. The investigated material (as SEM stub from each sample and subsamples of suspension) is curated in the collection of the Institute of Paleobiology, Polish Academy of Sciences, Warsaw, Poland, under the accession number ZPAL Pf.23.

## Results

### Systematic paleontology

Phylum Porifera Grant, 1836

Class Demospongiae Sollas, 1875

Subclass Heteroscleromorpha Cárdenas, Perez & Boury-Esnault, 2012

Order Spongillida Manconi & Pronzato, 2002

Family Spongillidae Gray, 1867

Genus *Ephydatia* Lamouroux, 1816

*Ephydatia* cf. *facunda* Weltner, 1895

Figures 2, 3, and 4

**Fig. 2:**
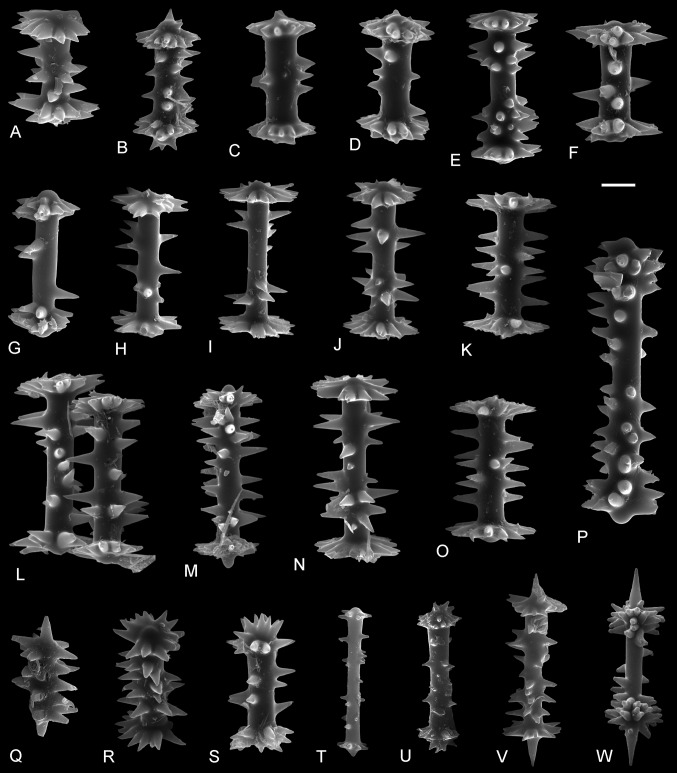
Gemmuloscleres of *Ephydatia* cf. *facunda* showing the range of morphological variability (strongly modified malformed birotules included). ZPAL Pf.23, SEM,* scale bar* 10 μm

**Fig. 3:**
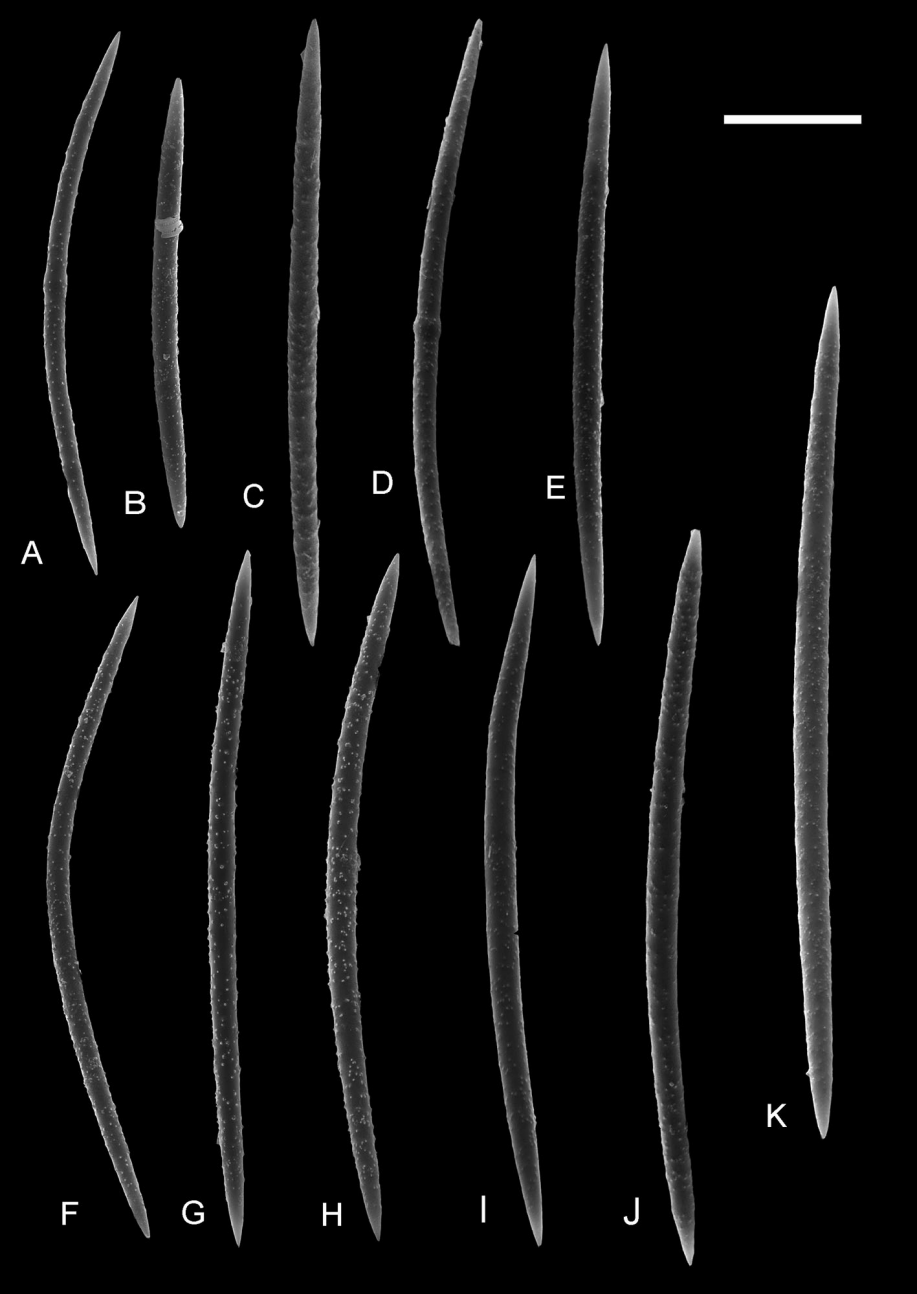
Megascleres belonging to *Ephydatia* cf. *facunda* from the Giraffe middle Eocene locality. ZPAL Pf.23, SEM, *Scale bar* 50 μm

**Fig. 4:**
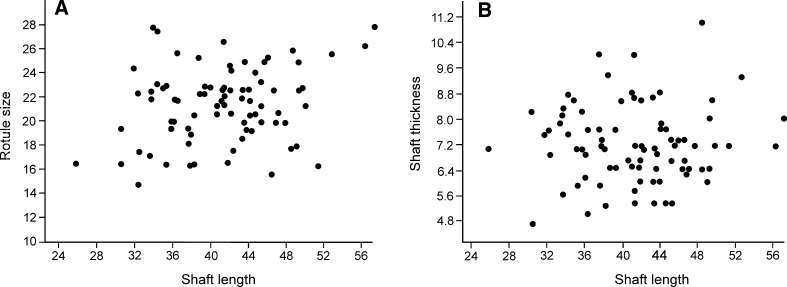
Gemmuloscleres morphometries of *Ephydatia* cf. *facunda*. Simple plots of rotule size versus length (**a**) and of shaft thickness of birotules versus length (**b**)

cf. 1895 *Ephydatia facunda* Weltner, p. 140–141.

cf. 1968 *Ephydatia facunda*—Penney and Racek, p. 92–93, pl. 7, figs. 16–19.

cf. 1979 *Ephydatia facunda*—De Rosa-Barbosa; p. 28–30, figs. 1–5.

non 2005 *Ephydatia facunda*—Manconi and Pronzato, p. 3243, fig. 3 [new species?].

cf. 2004 *Ephydatia facunda*—Pinheiro et al., p. 1072–1079, figs. 6–11 [cum syn.].

cf. 2007 *Ephydatia facunda*—Volkmer Ribeiro et al., fig. 3A.

cf. 2007 *Ephydatia facunda*—Volkmer Ribeiro and Machado, p. 164–166, figs. 4, 17–20.

*Material* Approximately 100 gemmuloscleres and 100 megascleres.

*Description* Only disassociated spicules have been found. Gemmuloscleres are birotules (Fig. 2) with incised margins of both rotules and spined shaft; spines are large and smooth with acute tips. The rotules are moderately smooth to strongly incised; at their center often rises a small rounded process (umbonate rotule). The number of spines on the shaft varies widely from 4 to about 20, but is usually 6–12; they are irregularly distributed and can extend in length to the rotule margin. The size of the gemmuloscleres is highly variable; length varies between about 26 and 57 μm, with an average of 41.5 μm; thickness of the shaft (not including spines) ranges from 5 to 11 μm, with an average of 7.2 μm; rotulae diameter ranges from 15 to 28 μm, with a mean of 21.4 μm. Some gemmuloscleres departing strongly from the typical morphology (Fig. 2P–W) are interpreted here as merely malformed (ecophenotypes), because similar morphs have been observed in Recent material (see De Rosa-Barbosa 1979). Possible megascleres (Fig. 3) of the sponge skeleton (disassociated monaxial spicules from the same sample) are large oxeas measuring 162.3–307 μm (the majority between 220 and 250 μm) in length, and are 8.7–12.8 μm (usually 9–10 μm) thick in the middle. Oxeas are straight to slightly curved, microspinose, with scattered small spines or tubercles, and the ends taper to a sharp point. Some are centrotylote.

*Remarks* The morphology of the gemmuloscleres is highly variable in our samples, and individual specimens from morphological extremes could be mistakenly considered to represent different species. Within any given sample, there are clear transitional forms between the morphological extremes of gemmuloscleres. There is also no pattern of change in the morphology of gemmuloscleres within the stratigraphical sequence, with a similar range of variability seen across the sequence. Statistical relationships between length of gemmuloscleres and rotule diameter (Fig. 4a) also do not show any spicular groupings. Therefore, we conclude that the spicules represent a single species.

We linked megascleres with the gemmuloscleres based on observations made for samples containing only *Ephydatia* gemmuloscleres. Our conclusion is that the slender oxeas with microspines and sharp tips belong to the same spicular complement of gemmuloscleres observed in the same samples (on the same SEM stub).

At present, only four fossil *Ephydatia* species are known, of which only two are well defined and described: *E. guttenbergiana* from the Eocene of Germany (Müller et al. 1982; Richter and Wuttke 1995) and *E. chileana* from the Miocene of Chile (Pisera and Saez 2003). The other two species, *E. kaiseri* (Rauff 1926) from the “pre-middle Eocene” of South Africa, and *E. fossilis* (Traxler 1894) from the Sarmatian (Miocene) of Hungary, are poorly circumscribed. Ampidiscs of the genus *Ephydatia*, without specific assignment, were also reported from the lower Oligocene of Germany (Martini and Schiller 1995). The taxon described here from the Giraffe locality differs considerably from all known fossil species. The most similar birotules in general shape and size are those of the species *E. chileana* from the Miocene of Chile (Pisera and Saez 2003), but the rotules of the Giraffe forms are significantly more incised. The size range and morphology of gemmuloscleres are similar in both species, but megascleres are clearly spinose in the Giraffe species, while those from the Chilean Miocene species are smooth and considerably shorter in comparison. The Giraffe fossil species is nearly identical to the recent species *Ephydatia facunda* as redescribed by De Rosa-Barbosa (1979) and Pinheiro et al. (2004) from Brazil (see also Nicacio and Pinheiro 2015). We have described it as *E.* cf. *facunda* due to the large time gap separating our material from the extant species. The species described as *E. facunda* from Cuba by Manconi and Pronzato (2005) has different gemmuloscleres from the type material from Brazil and represents a different species (Pinheiro et al. 2004). The megascleres that we attribute here to *E.* cf. *facunda* are morphologically very similar to those occurring in the holotype (De Rosa-Barbosa 1979), as well as newer material presented by Pinheiro et al. (2004). The megascleres from the Giraffe species are slightly smaller on average, but are within the range reported for *E. facunda* (De Rosa-Barbosa 1979; Pinheiro et al. 2004). In both cases the differences in spicule morphology are within the range often attributed to phenotypical plasticity relating to the environment (Poirrier 1974). The only other living species of *Ephydatia* known to have spines on the shaft is *E*. *robusta* (Potts 1888) from North America, but this species is often considered to be conspecific with *E*. *fluviatilis*. Very spiny shafts of birotules in the latter species are associated with harsh environmental conditions (high water temperatures, salty estuarine waters, and desiccation at gemmulation time) in Sardinian populations (RM, unpublished). *E. facunda* had not previously been recorded from the Nearctic Region (Manconi and Pronzato 2016), and today only two species of *Ephydatia*, *E. fluviatilis* and *E. muelleri*, occur in northern Canada (Ricciardi and Reiswig 1993). However, the gemmuloscleres of these latter species are easily differentiated from those of *E.* cf. *facunda* from the Giraffe locality.

## Discussion

*Ephydatia* is a very common, cosmopolitan genus known from all over the world except Antarctica. Six species have been recorded from the Northern Hemisphere, while only three species are known from the Southern Hemisphere (Manconi and Pronzato 2008). Due to the large variability in gemmulosclere morphology (Poirrier 1974), there is a lack of agreement regarding both the number of *Ephydatia* species in existence today and the range of variability within individual species. This makes studies of fossil material based on isolated gemmuloscleres especially difficult. The fact that representatives of this genus are often reported in the fossil record follows, without doubt, from the fact that its gemmuloscleres are very characteristic and easy to determine. This also reflects the fact that *Ephydatia* is a common, widespread, and euryoecious genus. Such a pattern of occurrence may be explained by its long evolutionary history that allowed for diversification and wide dispersal, a hypothesis supported by our findings.

Since *Ephydatia* spans cold to warm climates, it is difficult to use genus-level remains to infer temperature conditions in the Giraffe locality. However, if, as we believe, our material represents the tropical species *E. facunda*, then it does indeed suggest that this Arctic locality had a warm climate during the middle Eocene. Today, *E. facunda* is restricted to warm climates in Brazil and Argentina (Nicacio and Pinheiro 2015). In addition, other sponges occur in the same fossil samples (Pisera et al. 2013, 2014, and in prep.) and warm-water diatoms and synurophytes are also present (Siver and Wolfe 2009), supporting the hypothesis that the Giraffe locality was indeed significantly warmer than today during the middle Eocene.

In modern ecosystems, *Ephydatia facunda* is characteristic of freshwaters that are eutrophic, circumneutral, and mesohaline with abundant macrophytes (Volkmer-Ribeiro et al. 2004, 2007; Volkmer-Ribeiro and Machado 2007). However, there is no indication that the Giraffe lake ecosystem was ever a salty or brackish halobian environment, given that there are neither carbonates, evaporates, nor halophilous organisms anywhere in the sediments. Because middle Eocene forms of *E.* cf*. facunda* inhabited dilute and slightly acidic waters, as inferred from co-occurring algal microfossils (Wolfe et al. 2006; Siver and Wolfe 2009), the ecological tolerance of this sponge has either changed over time or is broader than previously envisaged. Alternatively, warm conditions may represent the dominant environmental control over the distribution of this species, with the capacity to override the influences of salinity and nutrients.

Furthermore, in some samples, there are spicules which resemble typical birotules of *E. facunda* but differ in size, the degree and character of rotule development, and the number of spines (Fig. 2P–W). Here, we treat them as ecophenotypes (malformed or teratological forms) of typical *E.* cf. *facunda* birotules rather than representatives of a clearly different taxon. Similar ranges of gemmulosclere morphology are observed in the type material (De Rosa-Barbosa 1979), whereas teratological *Ephydatia* gemmuloscleres have been reported in modern populations exposed to elevated trace metal concentrations (Poirrier 1974; Richelle-Maurer et al. 1994; Pisera and Saez 2003). One possibility is that teratological forms of *E*. cf*. facunda* in the Giraffe material have arisen because the taxon existed outside its ecological optimum with respect to salinity, as discussed above.

The well-preserved spicules of *Ephydatia* allow for a more precise comparison with extant species. The Giraffe gemmuloscleres fit unambiguously within the description of gemmuloscleres for *E. facunda* from Brazil. Our findings imply that the morphology of birotules is highly conserved and has undergone little (if any) evolutionary change since the middle Eocene. Spicular characters are also extremely well conserved in the marine sponge clades (for example: Łukowiak 2015). Similar results have been reported for euglyphid thecamoebans (Foissner and Schiller 2001; Barber et al. 2013), synurophytes (Siver and Wolfe 2005; Siver et al. 2013), and additional sponge groups such as the potamolepids (Pisera et al. 2013, 2014).

## Conclusions

Middle Eocene birotule gemmuloscleres from the Giraffe locality’s lake deposits in northern Canada have been identified as being very similar if not identical to gemmuloscleres of the extant species *Ephydatia facunda* Weltner (1895), but due to the large time gap separating them, they are described as deriving from *E*. cf. *facunda*. This finding represents yet another example of protracted evolutionary stasis with respect to the morphology of siliceous organisms, and of the expanded distribution of warm stenothermous taxa to the northern high latitudes during the Eocene greenhouse climate episode.

## References

[CR1] Barber A, Siver PA, Karis W (2013). Euglyphid testate amoebae (Rhizaria: Euglyphida) from an Arctic Eocene waterbody: evidence of evolutionary stasis in plate morphology for over 40 million years. Protist.

[CR2] Cárdenas P, Pérez T, Boury-Esnault N (2012). Sponge systematics facing new challenges. Advances in Marine Biology.

[CR3] De Rosa-Barbosa, R. 1979. Redescrição do tipo de *Ephydatia facunda* Weltner, 1895 (Porifera-Spongillidae). *Iheringia, Série Zoologia* 54: 27–34.

[CR4] Doria G, Royer DL, Wolfe AP, Fox A, Westgate JA, Beerling DJ (2011). Declining atmospheric CO_2_ during the Late Middle Eocene climate transition. American Journal of Science.

[CR5] Foissner W, Schiller W (2001). Stable for 15 million years: scanning electron microscope investigations of Miocene euglyphid thecamoebians from Germany, with description of the new genus *Scutiglypha*. European Journal of Protistology.

[CR6] Grant RE, Todd RB (1836). Animal Kingdom. Cyclopaedia of anatomy and physiology.

[CR7] Gray JE (1867). Notes on the arrangement of sponges, with the descriptions of some new genera. Proceedings of the Zoological Society London.

[CR8] Lamouroux, J.V.F. 1816. *Histoire des Polypiers Coralligènes Flexibles*, *vulgairement nommés Zoophytes*. Caen.

[CR9] Łukowiak M (2015). Reconstruction of the Late Eocene “soft” sponge fauna of southern Australia. Zootaxa.

[CR10] Manconi, R., and R. Pronzato. 2002. Spongillina n. suborder, Lubomirskidae, Malawispongiidae n. fam., Metaniidae, Metschnikowiidae, Paleospongillidae, Potamolepiidae, Spongillidae. In *Systema Porifera: a guide to the classification of sponges*, vol. 1, ed. H.J.N. Hooper, and R.W.M. Van Soest, 921–1019. New York: Kluwer Academic/Plenum.

[CR11] Manconi R, Pronzato R (2005). Freshwater sponges of the West Indies: discovery of Spongillidae (Haplosclerida, Spongillina) from Cuba with biogeographic notes and a checklist for the Caribbean area. Journal of Natural History.

[CR12] Manconi R, Pronzato R (2008). Global diversity of sponges (Porifera: Spongillina) in freshwater. Hydrobiologia.

[CR13] Manconi, R., and R. Pronzato. 2016. Chapter 3: Phylum Porifera. In *Thorp and Covich’s freshwater invertebrates. Keys to Nearctic fauna*, vol. II, 4th ed, ed. J. Thorp, and D.C. Rogers, 39–83. San Diego: Elsevier.

[CR14] Martini E, Schiller W (1995). Amphidisken der Schwammgattung *Ephydatia* im Unter-Oligozän von Sieblos/Rhön. Beiträge zur Naturkunde Osthessen.

[CR15] Müller WEG, Zahn RK, Maidhof A (1982). *Spongilla gutenbergiana* n. sp., ein Süßwasserschwamm aus dem Mittel-Eozan von Messel. Senckenbergiana Lethaea.

[CR16] Nicacio G, Pinheiro U (2015). Biodiversity of freshwater species (Porifera: Spongillina) from northeast Brazil: new species and notes on systematics. Zootaxa.

[CR17] Ott E, Volkheimer W (1972). *Paleospongilla chubutensis* n. g. et n. sp. ein Süsswasserschwamm aus der Kreide Patagoniens. Neues Jahrbuch für Geologie und Paläontologie Abhandlungen.

[CR18] Penney JT, Racek AA (1968). Comprehensive revision of a worldwide collection of freshwater sponges (Porifera: Spongillidae). Bulletin of the Museum of Natural History.

[CR19] Pinheiro US, Hajdu E, Correa MD (2004). First description of gemmules of *Ephydatia facunda* Weltner, 1895 (Porifera, Haplosclerida, Spongillidae) by scanning electron microscopy, with underwater observation of a large population from north-eastern Brasil. Journal of Natural History.

[CR20] Pisera A (2006). Palaeontology of sponges—a review. Canadian Journal of Zoology.

[CR21] Pisera, A. 2010. A high diversity Middle Eocene freshwater sponge fauna from the Giraffe Pipe crater lake, Canada. In *VIII World Sponge Conference 2010*, *Book of Abstracts*, Girona, Spain, September 20–24, 2010, 93.

[CR22] Pisera A, Saez A (2003). Paleoenvironmental significance of a new species of freshwater sponge from the Late Miocene Quillagua Formation (N Chile). Journal of South American Earth Studies.

[CR23] Pisera A, Siver PA, Wolfe AP (2013). A first account of freshwater potamolepid sponges (Demospongiae, Spongillina, Potamolepidae) from the middle Eocene: biogeographic and paleoclimatic implications. Journal of Paleontology.

[CR24] Pisera, A., P.A. Siver, and A. Wolfe. 2014. Middle Eocene lake deposits from the Giraffe pipe crater, northern Canada: a window on freshwater sponge evolution. In *4th International Palaeontological Congress. The history of life: a view from the Southern Hemisphere*, *abstract volume*, ed. C.V. Rubinstein, C.A. Marsicano, and B.G. Waisfeld, 595. Mendoza: CCT-CONICET.

[CR25] Poirrier MA (1974). Ecomorphic variation in gemmoscleres of *Ephydatia fluviatilis* Linnaeus (Porifera: Spongillidae) with comments upon its systematics and ecology. Hydrobiologia.

[CR26] Potts, E. 1888. Contributions towards a synopsis of the American forms of fresh-water sponges with descriptions of those named by other authors and from all parts of the world. *Proceedings of the Academy of Natural Sciences of Philadelphia* 39(1887): 158–279.

[CR27] Rauff H, Kaiser E (1926). Über prämitteleozäne fossilführende Süsswasser-Hornsteine aus der Namib. Die Diamantenwüste Südwest-Afrikas.

[CR28] Ricciardi A, Reiswig HM (1993). Freshwater sponges (Porifera, Spongillidae) of eastern Canada: taxonomy, distribution, and ecology. Canadian Journal of Zoology.

[CR29] Richelle-Maurer E, Degoudenne Y, Van de Vuver G, Dejonghe L, Van Soest RWM, van Kempen TMG, Braekman JC (1994). Some aspects of the ecology of Belgian freshwater sponges. Sponges in time and space.

[CR30] Richter G, Wuttke M (1995). Der Messel Süsswasser-Kieselschwamm *Spongilla gutenbergiana*, eine *Ephydatia*. Natur und Museum.

[CR31] Schindler T, Wuttke M, Poschmann M (2008). Oldest record of freshwater sponges (Porifera: Spongillina) spiculite finds in the Permo-Carboniferous of Europe. Paläontologische Zeitschrfit.

[CR32] Siver, P.A., J.M. Pelczar, A.M. Lott, and A. Pisera. 2010. The Giraffe Pipe database project: a web-based database for siliceous microfossils from a freshwater Eocene waterbody. In* Proceedings of the Seventh International Chrysophyte Symposium, New London, Connecticut, June 2008*. *Nova Hedwigia Beihefte* 136: 325–331.

[CR33] Siver PA, Wolfe AP (2005). Eocene scaled chrysophytes with pronounced modern affinities. International Journal of Plant Sciences.

[CR34] Siver PA, Wolfe AP (2009). Tropical ochrophyte algae from the Eocene of Northern Canada: a biogeographical response to past global warming. Palaios.

[CR35] Siver PA, Wolfe AP, Rohlf J, Shin W, Jo BY (2013). Combining geometric morphometrics, molecular phylogeny, and micropaleontology to assess evolutionary patterns in *Mallomonas* (Synurophyceae, Heterokontophyta). Geobiology.

[CR36] Sollas, W.J. 1875. Sponges. In* Encyclopedia Britannica*, 9th ed, 427–446. Edinburgh: Adam and Charles Black.

[CR37] Traxler L (1894). *Ephydatia fossilis*, eine neu Art der fossilen Spongilliden. Földatni Közlöny.

[CR38] Van Soest RWM, Boury-Esnault N, Vacelet J, Dohrmann M, Erpenbeck D, De Voogd NJ, Santodomingo N, Vanhoorne B, Kelly M, Hooper JNA (2012). Global diversity of Sponges (Porifera). PLoS One.

[CR39] Volkmer-Ribeiro C, Marques DM, De Rosa-Barbosa R, Machado VS (2004). Sponge spicules in sediments indicate evolution of coastal freshwater bodies. Journal of Coastal Research.

[CR40] Volkmer-Ribeiro C, Machado VS (2007). Freshwater sponges (Porifera, Demospongiae) indicators of some coastal habitats in South America: redescriptions and key to identification. Iheringia. Série Zoologia.

[CR41] Volkmer-Ribeiro C, de Ezcurra D, Parolin M (2007). Spicules of the freshwater sponge *Ephydatia facunda* indicate lagoonal paleoenvironment at the Pampas of Buenos Aires Province, Argentina. Journal of Coastal Research.

[CR42] Volkmer-Ribeiro, C., and J. Reitner. 1991. Renewed study of the type material of *Paleospongilla chubutensis* Ott and Volkheimer (1972). In *Fossil and recent sponges*, ed. J. Reitner, and H. Keupp, 121–133. Berlin: Springer.

[CR43] Weltner W (1895). Spongillidenstudien III. Katalog und Verbreitung der bekannten Süsswasserschwämme. Archiv für Naturgeschichte.

[CR44] Wolfe AP, Edlund MB, Sweet AR, Creithon SD (2006). A first account of organelle preservation in Eocene nonmarine diatoms: observation and paleobiological implications. Palaios.

